# A study of somatic *BRCA* variants and their putative effect on protein properties in malignant mesothelioma

**DOI:** 10.1515/pp-2023-0003

**Published:** 2023-03-24

**Authors:** Kritika Krishnamurthy, Kei Shing Oh, Sarah Alghamdi, Vathany Sriganeshan, Robert Poppiti

**Affiliations:** Department of Pathology, Montefiore Medical Center, Albert Einstein College of Medicine, Bronx, NY, USA; AM Rywlin Department of Pathology, Mount Sinai Medical Center, Miami Beach, FL, USA; Pathology, FIU/Herbert Werthein College of Medicine, Miami, FL, USA

**Keywords:** brca, *BRCA1*, *BRCA2*, disorder of proteins, malignant mesothelioma, somatic variants

## Abstract

**Objectives:**

The aim of this study is to analyze the prevalence of somatic mutations in *BRCA1* and *BRCA2* in malignant mesothelioma and their putative impact on protein properties.

**Methods:**

Eighteen cases of malignant mesothelioma were retrieved from the archives and for next generation sequencing analysis of *BRCA1* and *BRCA2* genes. Variants were analyzed using Ensembl VEP17, Polyphen 2.0 software, SIFT software, MutpredV2, and SWISS-MODEL homology-modeling pipeline server.

**Results:**

*BRCA2* variants were found in significantly higher percentage (22%) of cases (p=0.02). Five missense variants were identified. These were p.A2351P, p.T2250A, p.A895V, pG1771D, and p.R2034C. The SIFT scores of all except one were ≥ 0.03. The Polyphen scores of these four alterations were ≤0.899. In case of p.A2315, the SIFT score was 0.01, while the Polyphen 2 score was 0.921. MutPred2 scores were ≤0.180 for all. Loss of intrinsic disorder was predicted (Pr=0.32, p=0.07) for p.R2034C, while gain of intrinsic disorder was predicted for p.A2351P (Pr=0.36, p=0.01) and p.G1771D (Pr=0.34, p=0.02).

**Conclusions:**

*BRCA2* somatic variants were identified in 22% cases of malignant mesotheliomas in this study. The variants localize more frequently to the disordered regions of the protein and are predicted to affect the level of disorder.

## Introduction

Malignant mesothelioma is a malignancy that develops from the mesothelial cells lining the pleura, peritoneum, pericardium, and tunica vaginalis of the testes [1]. Malignant mesotheliomas are generally very aggressive with poor prognosis and survival [2, 3]. They display a high rate of chemoresistance both innate and acquired [4, 5]. The global mortality from malignant mesothelioma is estimated to be around 9.9 per million with large regional variations [6].

Based on histopathology, malignant mesothelioma has three main morphological subtypes, epithelioid, sarcomatoid, and the biphasic pattern which has both the subtypess admixed in various proportions. The sarcomatoid variant is rare and portends a poorer prognosis [7].

Malignant mesothelioma of pleura and peritoneum show a strong association with asbestos exposure [8]. Airborne exposure to asbestos both in the form of short term, heavy occupational exposure, as well as chronic, low-level environmental exposure has been shown to portend an increased risk for developing malignant mesothelioma. However, not all exposed individuals develop the disease [9] and not all cases of malignant mesothelioma can be traced to asbestos exposure [10]. This is further highlighted by the lack of a corresponding decrease in the incidence of malignant mesothelioma, despite a substantial and successful global effort, including introduction of strict legislature and mining regulations, to limit asbestos exposure [11], [12], [13]. Although there is a long latency between asbestos exposure and diagnosis of malignant mesothelioma, the current data taken together with the lack of any uniformity in dose-response relationship between the disease and asbestos exposure, point to the strong role of an underlying inherited susceptibility.

Germline mutations in BAP1 have been shown to have an established temporal association with malignant mesothelioma [14, 15]. Recently germline mutations in cancer susceptibility genes including *ATM*, *CDKN2A*, *BRCA1*, *BRCA2*, *MSH6*, *MLH1*, *PALB2*, and *TP53*, have been reported in some cases of malignant mesothelioma [8, 16]. However, the prevalence and significance of somatic mutations in known cancer susceptibility genes in malignant mesothelioma remain elusive. The aim of this study is to analyze the prevalence and frequency of somatic mutations in well-known cancer susceptibility genes, *BRCA1* and *BRCA2* in a small cohort of malignant mesothelioma patients and explore its putative impact on protein properties contributing to cancer pathogenicity.

## Materials and methods

This study was approved by the Institutional Review Board and Ethics Committee of Mount Sinai Medical Center, Miami Beach, Florida, and was carried out in collaboration with Quest Diagnostics.

### Case selection

Eighteen cases of malignant mesothelioma of the pleura and the peritoneum were retrieved from the pathology archives. Patients with other prior or concomitant malignant neoplasms were not included in this study. Pertinent case data including stage, grade, histological subtype, and the presence and location of metastasis were recorded.

H and E slides were examined to identify blocks with adequate tumor content. Ten unstained sections were cut from the selected formalin-fixed paraffin embedded tissue blocks.

### Genetic testing and data analysis

The unstained sections along with representative H and E slides were sent to Quest Diagnostics for next generation sequencing analysis of *BRCA1* and *BRCA2* genes.

Statistical analysis was performed using IBM SPSS 26 software (Armonk, NY, USA). Nonparametric data was assessed for statistical significance using Fisher exact and chi square tests.

### Variant interpretation

Each variant detected was crosschecked using Ensembl VEP [17, 18] and if available, COSMIC [19]. Polyphen 2.0 software version 2.2.2 and SIFT software version 5.2.2 were used to predict the functional effect of each single nucleotide polymorphism (SNP) [20, 21]. Allele frequency was derived from gnomAD (The genome aggregation database) [22]. Mutpred V2 software [23] was used to predict the effect of individual SNPs on motif, structure and dynamics and protein binding. P value of less than 0.10 was considered significant in terms of predicted protein alterations. The SWISS-MODEL homology-modeling pipeline server [24] was used to predict the tertiary structure of modified proteins using template ID P51587 (1iyj.1.B).

## Results

A total of eighteen cases of malignant mesothelioma were analyzed for genetic alterations in *BRCA1* and *BRCA2* genes in this study. The average age of the study population was 77.6 ± 10.5 years. The subjects included four women and fourteen men. The primary site of the malignant mesothelioma was isolated to the pleura in twelve cases, peritoneum in five cases and pericardium in one case. Clinically, thirteen patients had stage 4 disease, while one each had stage 3 and stage 2, and two had stage 1 disease, at the time of specimen retrieval. One was a recurrence of a previously treated peritoneal mesothelioma.

Based on histology, fourteen of the malignant mesotheliomas had epithelioid morphology, while three were biphasic and one was sarcomatoid. All but two of the eighteen patients were still alive and in various stages of treatment and follow up at the time of this study. Characteristics of the study subjects are summarized in Table 1. The average tumor percentage as seen on light microscopy on the H and E slide was 46.8 ± 15.7%.

**Table 1:** Characteristics of study subjects.

n		18
Age, years (mean ± SD)		77.6 ± 10.5
Gender	M	14
	F	4
Primary site	Pleura	12
	Peritoneum	5
	Pericardium	1
Stage^a^	I	2
	II	1
	III	1
	IV	13
Presence of metastasis	Y	13
	N	5
Morphology	Epithelioid	14
	Sarcomatoid	1
	Biphasic	3
Tumor percentage		46.8 ± 15.7%
Mutation	*BRCA1*	1
	*BRCA2*	4
	Absent	13

^a^One case was a recurrence and was not allotted a stage. *BRCA 1* and *BRCA 2* gene mutations.

Of the eighteen cases tested, *BRCA1* variant was found in only one case (5.5%). *BRCA1* variant, was a novel missense variant, c.3155 A>G/p.N1052S, at a variant allele frequency of 45.2%. The SIFT score was 0.06 (tolerated) while polyphen score was 0.829 (possibly damaging). The MutPred2 score was 0.059 reflecting likely lack of pathogenicity. The population allele frequency was less than 0.005 for all variants. As such, the variant was classified as variant of uncertain significance.

On the other hand, *BRCA2* variants were found in 4 cases (22%) which was significantly higher than the frequency of *BRCA1* variants (p=0.02). A total of six variants which were identified, at variant allele frequency ranging from 4.9 to 76.9%, all of which led to missense proteins. The resulting substitutions were p.A2351P, p.T2250A, p.A895V, pG1771D and p.R2034C. Only p.R2034C was seen in two cases. The SIFT scores of all except the first substitution were ≥0.03 reflecting a tolerated nature. Similarly, the Polyphen scores in these four alterations were ≤0.899 reflecting benignancy. In case of p.A2315, the SIFT score was 0.01, while the Polyphen 2 score was 0.921, both indicating a deleterious and probably damaging nature. Variant data along with allele frequency, Polyphen 2 and SIFT scores summarized in Table 2.

**Table 2:** *BRCA2* variant details with SIFT and polyphen scores.

Chr13 position	Ref allele	Variant allele	Zygosity	Variant frequency	Aminoacid change	Populaiton frequency GenomAD	RS identifier	SIFT score	Polyphen score
32,911,176	C	T	Het	4.9	p.Ala895Val	NA	NA	0.12	0.209
32,913,804	G	A	Het	37.1	p.Gly1771Asp	0.000335	rs80358755	0.65	0.072
32,914,592	C	T	Het	76.9	p.Arg2034Cys	0.003126	rs1799954	0.03	0.54
32,914,592	C	T	Het	60.2	p.Arg2034Cys	0.003126	rs1799954	0.03	0.54
32,915,240	A	G	Het	27.8	p.Thr2250Ala	0.000111	rs80358899	0.06	0.019
32,929,041	G	C	Het	52.9	p.Ala2351Pro	0.000008	rs80358930	0.01	0.921

However, the MutPred2 scores were ≤0.180 for all. None of the predicted ELM or PROSITE motif alterations met the threshold for statistical significance. Loss of intrinsic disorder was predicted (Pr=0.32, p=0.07) for p.R2034C, while gain of intrinsic disorder was predicted for p.A2351P (Pr=0.36, p=0.01) and p.G1771D (Pr=0.34, p=0.02). In addition, an alteration of coiled coil was predicted for p.A2351P (Pr=0.02, p=0.10) and p.G1771D (Pr=0.04, p=0.07). A gain of B-factor was also predicted for p.A2351P (Pr=0.23, p=0.09). None of the other protein properties included in the MutPred algorithm met the threshold for statistical significance. The Mutpred2 predictions are summarized in Table 3.

**Table 3:** Impact of missense mutations on protein property as predicted by MutPred2.

Aminoacid change	Loss of intrinsic diorder		Gain of intrinsic diorder		Alteration of coil		Gain of B-factor		MutPred2 score
	Pr	P	Pr	P	Pr	P	Pr	P	
p.Ala895Val									0.012
p.Gly1771Asp			0.34	0.02	0.04	0.07			0.024
p.Arg2034Cys	0.32	0.07							0.019
p.Thr2250Ala									0.012
p.Ala2351Pro			0.36	0.01	0.02	0.1	0.23	0.09	0.03

**Figure 1:**
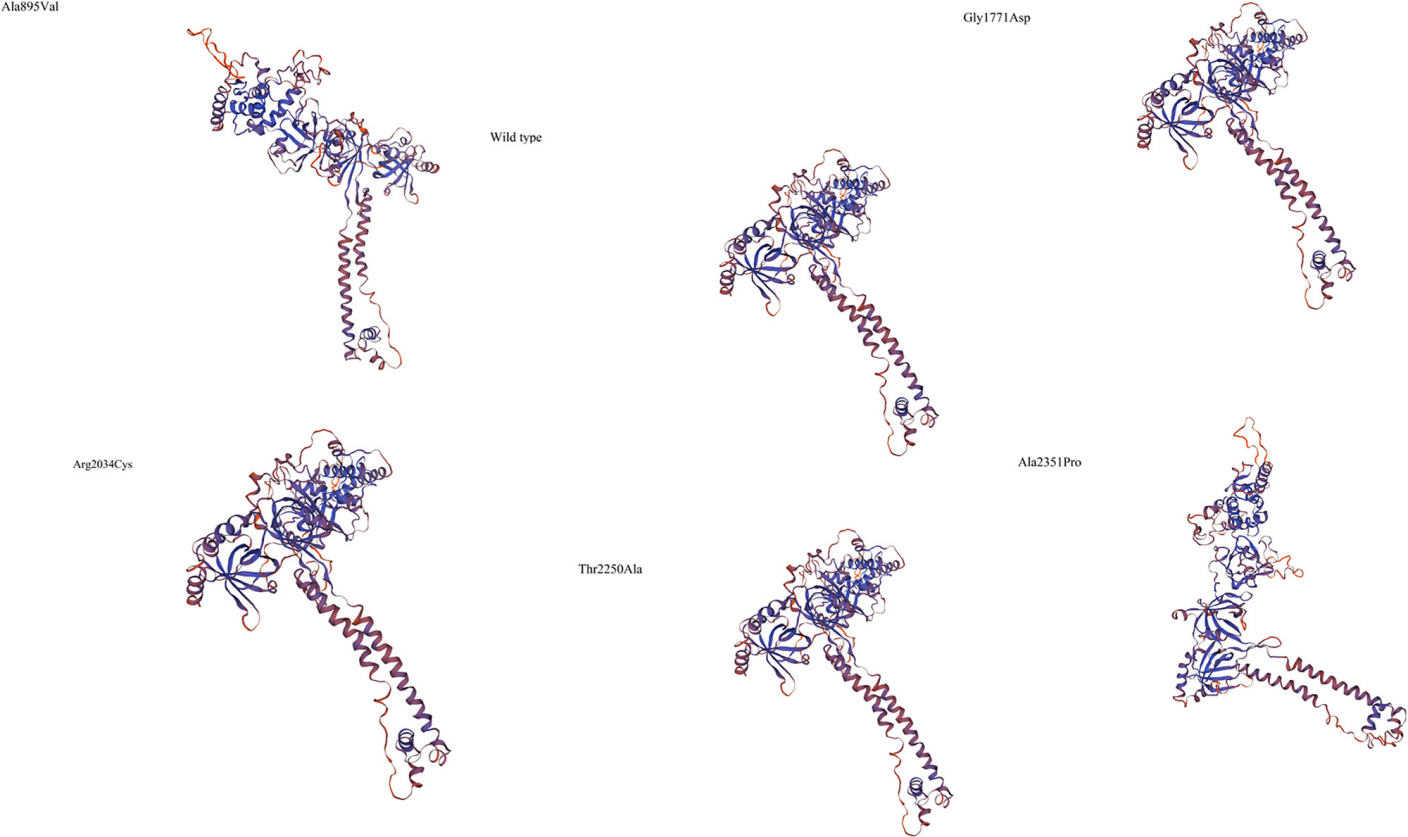
Static images of 3D models built using SWISS-MODEL server.

The homology modeling scores as predicted by the SWISS-MODEL server are summarized in Table 4 and static images of each model is seen in Figure 1.

**Table 4:** Homology modeling scores predicted by SWISS-MODEL server.

Variant	MolProbity score	Clash score	Ramachandran score	Q mean value
WT	3.21	14.9	73.52	0.7
Ala895Val	3.21	14.9	73.52	0.7
Gly1771Asp	3.21	14.9	73.52	0.7
Arg2034Cys	1.98	0	81.63	0.51
Thr2250Ala	1.98	0	81.63	0.51
Ala2351Pro	3.21	14.9	73.52	0.7

No statistically significant associations were found between any of the *BRCA1* or 2 variants and any of the demographic, histologic or clinicopathologic characteristics in this small cohort.

## Discussion

*BRCA1* and *BRCA2*, discovered in 1990s, are known cancer predisposition genes. *BRCA1* is located on chromosome 17 and codes for a 5.6 kb protein while *BRCA2* is located on chromosome 13 and codes for a 10.2 kb protein [23]. BRCA genes are intrinsically expressed in a wide variety of tissue and are the integral components of the homologous DNA repair pathway. In addition, BRCA genes are essential for transcriptional regulation, cell growth and genomic integrity [24]. The complexity of these large proteins taken together with the plasticity allows the possibility of additional roles for BRCA proteins that remain elucidated. For instance, the role of *BRCA1* as a nuclear-cytoplasmic transfer protein though well documented, is yet to be explained [25].

Germline mutations in BRCA genes are well established risk factors for ovarian and breast cancers and inherited cancer syndromes [23]. In addition to elevated cancer risk, germline *BRCA1* or *BRCA2* mutations in breast, ovarian and prostate cancer, predict both a better prognosis and superior response to cisplatin-based chemotherapy [26]. These patients have also demonstrated sensitivity to treatment with poly (ADP-ribose) polymerase inhibitors (PARPi) [27], [28], [29]. Similar to these *BRCA1* and *BRCA2* associated malignancies, malignant mesothelioma patients with germline BRCA mutations are reported to have improved survival and better response to cisplatin-based regimens as well as benefit from PARPi therapy [30, 31]. A recent phase II clinical trial (MiST1 trial) showed promising results with PARPis in mesothelioma patients [32].

Panou et al., in their targeted capture and next generation sequencing study of 85 cancer susceptibility genes on germline DNA from 198 malignant mesothelioma patients, found *BRCA1* germline mutations in 0.0051 proportion of mesothelioma patients included in their study compared to 0.002 estimate in the non-cancer population (p>0.05). In the same study, *BRCA2* germline mutations were seen in 0.0152 proportion of mesothelioma patients as compared to the 0.003 estimate in non-cancer population (p=0.03) [10]. This translates to a prevalence of 8.1% for *BRCA2* variants in malignant mesothelioma compared to 2.1% for *BRCA1* in this US based study. Hiltbrunner et al., in a much larger study based out of Europe, found germline alterations in *BRCA2* in 2.3% of malignant mesothelioma cases, with a slightly higher prevalence in pleural mesotheliomas (2.5%) as compared to peritoneal mesotheliomas (1.7%) [33]. Both the studies had a predominance of pleural mesotheliomas, thus the variation in prevalence of BRCA alterations is probably attributable to population and environmental factors. However, as seen in both studies, *BRCA2* is altered more frequently than *BRCA1* in malignant mesothelioma.

Although the clinical significance of germline BRCA mutations in several cancers including malignant mesothelioma is well established [15, 16], the implications of somatic BRCA mutations is yet to be elucidated.

Several studies have reported somatic BRCA mutations in ovarian cancers, which remain the preferred study model for BRCA alterations. The cancer genome atlas research network reported somatic BRCA mutations of 3% in their whole exome sequencing of 316 high grade serous cancers of the ovary and the matched normal DNA [34]. Though various studies report differing prevalence rates of BRCA somatic mutations in ovarian cancers, on an average BRCA somatic mutations (no germline BRCA mutations) occur in approximately 5–7% of ovarian cancer cases [34, 35]. Ledermann et al. in a randomized, double blind, placebo controlled study for evaluation of Olaparib found 14% of patients to have somatic BRCA mutations (without germline mutations). This subgroup of patients with exclusively somatic BRCA mutations had fewer progression events in the Olaparib arm of the trial as compared to the placebo arm [36]. Although the number of patients with exclusively somatic BRCA mutations analyzed is low, these findings suggest that Olaparib is effective in tumors with somatic BRCA mutations and ongoing prospective studies including SOLO-2, NOVA and ARIEL will shed further light on these results [36].

Given the growing importance of somatic BRCA mutations in ovarian carcinomas, the frequency and functional effects of somatic BRCA mutations in other BRCA associated cancers need to be clarified. This study is amongst the first to elaborate on somatic BRCA mutations in malignant mesothelioma. The distribution of somatic BRCA mutations seen in this study mirrors the findings in germline BRCA with a higher frequency of *BRCA2* mutations as compared to *BRCA1* mutations [10, 33, 37]. The *BRCA2* somatic alterations were limited to single nucleotide polymorphism in all cases similar to pre viously reported germline findings [10]. Though there was no statistically significant difference in survival between the *BRCA2* mutant and non-mutant groups, all the BRCA mutant patients were alive at the time of the study as compared to the nonmutant group in which two patients had died. The study sample was small and that might explain the lack of statistical significance for this finding.

Loss or gain of intrinsic disorder was the most common predicted effect seen in three of the SNPs identified in this study. Protein disorder allows for structural and binding site promiscuity allowing for varied protein interactions and functions in a single polypeptide. Disorder seems to be intrinsic to function and is highly prevalent in proteins involved in signal transduction, transcription, DNA repair and chromatin remodeling, thus enabling transient binding with multiple partners. Presence of amino acids such as Arg, Pro, Gln, Gly, Glu, Ser, Ala, and Lys, often in repeats, and absence of structure promoting amino acids Trp, Cys, Tyr, Ile, Phe, Val, Asn, and Leu results in intrinsic disorder [38]. All missense mutations seen in *BRCA2* in this study resulted in replacement of disorder promoting aminoacids in all cases but one in which a disorder neutral aminoacid was replaced by a disorder promoting one.

The *BRCA2* protein lacks a stable secondary structure. This structural plasticity of *BRCA2* protein is attributable to the intrinsically disorder regions that are a hallmark of cancer associated proteins [39, 40]. The disordered regions include the recombinase RAD51 binding site as well as other short regions with intermediate disorder propensity [41]. Recently characterized conserved fragments among the disordered regions include BRCA248–284, BRCA2250–2500, BRCA21093–1158, and BRCA22213–2342 [42]. Two of the missense mutations found in this study lie within these well characterized regions. However, several other disordered, conserved motifs have been demonstrated based on predictive analysis but as of yet remain uncharacterized [42].

For instance, the BRCA2G1771D, where the small, aliphatic glycine is replaced by negatively charged and bulky glutamate leads to steric hinderance in this BRC repeat region [43]. When imaged for oligomerization and solidity, in the presence and absence of RAD2, analysis found that though in the absence of RAD51, BRCA2G1771D formed irregular multimeric assemblies with extended shape and average solidity, like *BRCA2* wildtype and deviated only marginally in oligomerization. In the presence of RAD51, though the conformational distribution was similar, the molecules with decreased solidity were higher at 4% in BRCA2G1771D as compared to 1% in *BRCA2* wildtype [43]. However, the significance of this structural difference is as yet unknown and deciphering it is beyond the scope of this study. With evolution of the understanding of these intrinsically disordered regions, and their multitude functions, several of the variants that are of uncertain significance may gain clinical significance and will have to be reclassified differently.

*BRCA2* is integral in DNA replication, transcription and repair, and the disordered regions of *BRCA2* contribute significantly in these mechanisms, many of which are still emerging. Defects in any of these may promote chromosomal instability and tumorigenesis [44]. With growing evidence supporting the predictive role of somatic *BRCA2* mutations in the effectiveness of PARPi therapy, the potential role of somatic *BRCA2* mutations is expanding. Because of the promising results of PARPi therapy in BRCA (germline) mutated malignant mesotheliomas, the considerable frequency of *BRCA2* somatic variants in malignant mesotheliomas found in this study and the putative effect of these variants on protein properties including intrinsic disorder, these findings merit further evaluation in a larger cohort.

## Conclusions

In the light of growing significance of somatic BRCA mutations, this study investigated the frequency of somatic *BRCA1* and *BRCA2* variants in a small cohort of malignant mesothelioma patients and found *BRCA2* somatic variants to be much more common than *BRCA1* variants. The *BRCA2* variants found in this study set localize more frequently to the disordered regions of the protein and are predicted to alter the level of disorder. These finding are significant and merit further investigation of somatic *BRCA2* variants in a larger cohort.
